# Influence of airway management strategy on "no-flow-time" during an "Advanced life support course" for intensive care nurses – A single rescuer resuscitation manikin study

**DOI:** 10.1186/1471-227X-8-4

**Published:** 2008-04-10

**Authors:** Christoph HR Wiese, Utz Bartels, Alexander Schultens, Tobias Steffen, Andreas Torney, Jan Bahr, Bernhard M Graf

**Affiliations:** 1 Department of Anaesthesiology, Emergency and Intensive Care medicine, University of Göttingen, Germany; 2 Department of Anaesthesiology, Klinikum Wolfenbüttel, Germany; 3 Department of Anaesthesiology, Klinikum Braunschweig, Germany

## Abstract

**Background:**

In 1999, the laryngeal tube (VBM Medizintechnik, Sulz, Germany) was introduced as a new supraglottic airway. It was designed to allow either spontaneous breathing or controlled ventilation during anaesthesia; additionally it may serve as an alternative to endotracheal intubation, or bag-mask ventilation during resuscitation. Several variations of this supraglottic airway exist. In our study, we compared ventilation with the laryngeal tube suction for single use (LTS-D) and a bag-mask device. One of the main points of the revised ERC 2005 guidelines is a low no-flow-time (NFT). The NFT is defined as the time during which no chest compression occurs. Traditionally during the first few minutes of resuscitation NFT is very high. We evaluated the hypothesis that utilization of the LTS-D could reduce the NFT compared to bag-mask ventilation (BMV) during simulated cardiac arrest in a single rescuer manikin study.

**Methods:**

Participants were studied during a one day advanced life support (ALS) course. Two scenarios of arrhythmias requiring defibrillation were simulated in a manikin. One scenario required subjects to establish the airway with a LTS-D; alternatively, the second scenario required them to use BMV. The scenario duration was 430 seconds for the LTS-D scenario, and 420 seconds for the BMV scenario, respectively. Experienced ICU nurses were recruited as study subjects. Participants were randomly assigned to one of the two groups first (LTS-D and BMV) to establish the airway. Endpoints were the total NFT during the scenario, the successful airway management using the respective device, and participants' preference of one of the two strategies for airway management.

**Results:**

Utilization of the LTS-D reduced NFT significantly (p < 0.01). Adherence to the time frame of ERC guidelines was 96% in the LTS-D group versus 30% in the BMV group. Two participants in the LTS-D group required more than one attempt to establish the LTS-D correctly. Once established, ventilation was effective in 100%. In a subjective evaluation all participants preferred the LTS-D over BMV to provide ventilation in a cardiac arrest scenario.

**Conclusion:**

In our manikin study, NFT was reduced significantly when using LTS-D compared to BMV. During cardiac arrest, the LTS-D might be a good alternative to BMV for providing and maintaining a patent airway. For personnel not experienced in endotracheal intubation it seems to be a safe airway device in a manikin use.

## Background

The European Resuscitation Council (ERC) released a revised edition of their resuscitation guidelines in November 2005 [1]. Following important adjustments were incorporated:

- emphasis on chest compression.

- reduction of no-flow-time (NFT), the time during which no chest compression occurs.

- defibrillation: following each 2-minute cycle of CPR only one electric shock should be applied, for shockable rhythms.

- expansion of airway management options.

Endotracheal intubation (ET) remains the gold standard of airway management [1]. No more than thirty seconds should be used for attempting tube placement, and only personnel familiar with this procedure should use it to reduce the possibility of unrecognised oesophageal intubation and accidental hypoxia. Since the majority of emergency medicine personnel are unfamiliar with ET, alternative airway management options are required during resuscitation. Bag-mask-ventilation (BMV) as the least invasive procedure is certainly the most obvious. Other airway management options stated in the ERC guidelines are:

- the laryngeal mask (LMA).

- the laryngeal tube (LT).

- the combitube (CT).

Alternative airway devices for use during resuscitation should meet the following criteria: They should be easy to handle by persons not trained in their use, and they should guarantee reasonable protection against aspiration [1].

In 1999, the LT was introduced as an alternative for protecting the difficult airway [2-6]. Especially the LT version with a gastric suction option (LT-S, introduced in 2002) meets the above mentioned criteria, as recent studies suggest [7]. In addition there is a growing interest in emergency use of an airway device that was originally developed for use in anaesthesia [4-6].

The aim of this manikin study was to show the effect on NFT using the LTS-D, and the efficacy of the LTS-D in managing the airway used by ICU nurses during a cardiac arrest scenario compared to BMV.

## Methods

Study subjects were recruited from the participants of a one day ALS-course designed for ICU nurses, after obtaining written informed consent. The study was conducted following completion of the course. Simulated scenarios with a resuscitation manikin (Laerdal™ "Resusci Anne Advanced Skilltrainer"; Laerdal Inc., Norway) required participants to resuscitate a patient with ventricular fibrillation (VF) in a team consisting of two rescuers. The duration of the scenario was limited to 430 seconds using the LTS-D, and 420 seconds using BMV (Table 1). Participants were randomly assigned to one of the two groups first (LTS-D and BMV) to establish the airway. All participants completed both scenarios. The LTS-D was used in the first scenario and BMV in the second.

**Table 1:** Scenario target times

**Activity**	**LTS-D time (total time)**	**BMV time (total time)**
Time to check the victim for a response	15 s (15 s)	15 s (15 s)
LTS-D placement after first thirty chest compressions	10 s (25 s)	0 s (15 s)
1st whole CPR cycle	120 s (145 s)	120 s (135 s)
1st defibrillation	15 s (160 s)	15 s (150 s)
2nd CPR cycle	120 s (280 s)	120 s (270 s)
2nd defibrillation	15 s (295 s)	15 s (285 s)
3rd CPR cycle	120 s (415 s)	120 s (405 s)
3rd defibrillation	15 s (**430 s**)	15 s (**420 s**)

Fifty participants took part in this prospective study. Inclusion criteria were first ALS-course according to the ERC 2005 guidelines and unfamiliarity with the LTS-D. To reduce interobserver bias, courses were held by the same instructors.

Following demographic data were obtained and compared:

• age.

• gender.

• years experience on ICU.

• previous ALS courses which were not according to the ERC 2005 guidelines.

Study end points were:

Primary:

• NFT.

• time to first ventilation (LTS-D vs. BMV).

Secondary:

• Total ventilation time. Sum of individual ventilations with BMV, and LTS-D (including time of LTS-D placement), respectively.

• time of defibrillation during the scenario.

• time until unconsciousness was diagnosed.

• participants' retrospective subjective evaluation of both airway management types (collected by questionnaire).

Data were recorded on the computer attached to the resuscitation manikin using manufacturer's software. In addition, each scenario was filmed on video to allow for retrospective analysis. The statistics programme SPSS 12.0 (SPSS Inc.) was employed and the Wilcoxon signed ranks test used. A p-value <0.05 was considered significant.

According to the declaration of Helsinki [8] data were made anonymous, thus tracing data to individual participants was made impossible.

To ensure the reliability of comparing times, the following set up was chosen: Two participants were involved in each scenario. The first was designated as the main rescuer; the other was called secondary rescuer. Main rescuers were responsible for ventilation, establishing airway device and chest compression in the scenario. Therefore the scenario was designed as a single rescuer resuscitation. The secondary rescuer assisted the main rescuer in all ancillary activities. Each participant was assigned to the main and secondary position twice.

The main rescuer had to place LTS-D after confirming unconsciousness and the first thirty chest compressions; unsuccessful in the first attempt, the rescuer had to re-attempt insertion after first defibrillation and the following thirty chest compressions. Until achieving successful placement/insertion of the LTS-D, the rescuer had to ventilate the manikin using BMV. Successful placement/insertion of the LTS-D was defined as the ability to reach a tidal volume of 400–600 ml for single ventilation of the manikin.

NFT was defined as time since during no chest compressions and hence no organ perfusion occurs. The following NFTs were unavoidable in the standardised scenario utilized in this study:

• 15 seconds to diagnose unconsciousness.

• 10 seconds for insertion of the LTS-D (following airway check).

• 10 seconds for respiration during each cycle of CPR.

• 15 seconds for each defibrillation with a semi AED (CorPuls 08/16™).

Times required for each defibrillation were due to the time required by the semi-automated defibrillator (CorPuls 08/16™) for analysis and shock. Remaining times in the scenarios are in accordance with the ERC guidelines 2005 [1].

Total respiration time was defined as time which the rescuer needed to ventilate the manikin (sum of time for single ventilation with airway device), including the insertion of the LTS-D, and each placement of the bag-mask. Average ventilation time was defined as the time required for every single ventilation of the manikin.

## Results

The study included fifty participants. Demographic data are presented in Table 2. All participants were active ICU nurses.

**Table 2:** Demographic data

**Gender**	**Male**	**Female**				
	19 (38%)	31 (62%)				
**Vocational experience (years)**	**<5**	**6–10**	**11–15**	**>15**		
	6 (12%)	18 (36%)	5 (10%)	21 (42%)		
**Age (years)**	**18–25**	**26–30**	**31–35**	**36–40**	**41–45**	**>45**
	4 (8%)	14 (28%)	10 (20%)	11 (22%)	10 (20%)	1 (2%)
**Number of previous ALS courses which were not according to ERC 2005 guidelines**	**0**	**1**	**>1**			
	35 (70%)	14 (28%)	1 (2%)			

### NFT and defibrillation

NFT in the LTS-D group was significantly (p < 0.01) shorter than in the BMV group; 105.2 s (range 94–124 s), versus 149.7 s (range 124–179 s), respectively. This translates into an NFT fraction of 0.24 (LTS-D), and 0.36 (BMV) of each scenario.

Table 3 summarises the times required to, as well as the total number of, defibrillations in each group.

**Table 3:** Defibrillations during the scenarios

**Defibrillation**	**Time-to-shock [„LTS-D“] seconds (min/max)**	**Time-to-shock [„BMV“] seconds (min/max)**	**p-value**	**Number of participants that completed this cycle [„LTS-D“]**	**Number of participants that completed this cycle [„BMV“]**	**p-value**
1. Defibrillation	152,3 (119,4–192,3)	162,9 (120,1–200,3)	>0.05	50 (100%)	50 (100%)	>0.05
2. Defibrillation	282,6 (230,5–340,7)	311,4 (265,8–365,3)	>0.05	50 (100%)	50 (100%)	>0.05%
3. Defibrillation	407,4 (362,5–419,8)	412,3 (380,2–420,0)	>0.05	48 (96%)	15 (30%)	**<0.05**

Significantly more participants achieved three defibrillations in the LTS-D group (48; 96%) than in the BMV group (15; 30%; p < 0.05) within the set time frames. In the LTS-D group 48 participants (96%) adhered to the ERC guidelines 2005, including the time frame. Using BMV for airway management fifteen participants (30%) adhered to the ERC 2005 guidelines.

In both groups there was no significant difference concerning defibrillation times during the scenario (p > 0.05; Table 3).

### Using the LTS-D for airway management

Ninety-six percent of the participants successfully insert the LTS-D on first attempt; four percent required a second attempt. Adequate tidal volumes were achieved through the LTS-D (400–600 ml) in 100%.

Time to first respiration using the LTS-D was 25.1 seconds after starting the scenario (range 22–39 s). This time included the time to confirm unconsciousness of the manikin, the time to place the LTS-D, but excluded the time for the first thirty chest compressions after confirming cardiac arrest. Including the first thirty chest compressions the time to first respiration using the LTS-D was 39.3 seconds (range 31–53 s). The time to insert the LTS-D required an average of 12.9 seconds (range 10–27 s).

Primary BMV was not intended in this scenario. Single ventilation averaged a time of 1.2 seconds (this included inspiration and expiration of the manikin; range 1–1.6 s). The ERC defined to give each rescue breath about 1 s with enough volume to make the victim's chest rise.

Total respiration times averaged 48.6 seconds (range 39–59 s). This time included the time for every single breath and the correct insertion of the LTS-D in the manikin.

### Using BMV for airway management

Time to first respiration in the BMV group was 13.2 seconds (range 9–18 s) after starting the scenario. This included the time to confirm unconsciousness of the manikin, and excluded the time for the first thirty chest compressions given before first ventilation was started. Including the first thirty chest compressions the time to first respiration using the BMV was 23.4 seconds (range 19–35 s).

Each single ventilation of the manikin averaged 3.8 seconds (range 1.9–5.3 s). This included inspiration and expiration of the manikin.

Total respiration time amounted to 107.3 s (range 57–158 s). This time included the placement of the face-mask and the self-inflating bag.

Twelve participants (24%) attained effective tidal volumes of 400–600 ml with BMV. The majority of participants were unable of generating efficient ventilation.

### Questionnaire

At the end of this manikin study all participants favoured the utilization of the LTS-D in a cardiac arrest scenario. The main reason was that handling of the LTS-D was considered easier to learn than bag-mask ventilation, resulting in more confidence.

## Discussion

The ERC 2005 guidelines aim at reducing NFT since during this time no chest compressions and hence no organ perfusion occurs. In our manikin study this time adds up a total minimal NFT of 100 seconds (LTS-D), and 90 seconds (BMV). The NFT using the LTS-D was 105.2 s (24.5% of total time, range 94–124 s), and significantly less (p < 0.01) than the NFT utilizing the BMV, with 149.7 s (35.6% of total time, range 124–179 s).

Using the LTS-D for airway management in a manikin study the participants approximately met the guideline NFT criteria [1]. No participant was able to achieve this with BMV. Moreover, only 30% of the skilled ICU nurses were able to apply three defibrillations within the time-frame of the ERC guidelines, while on the other hand 96% of participants' fulfilled to the ERC guidelines, including three defibrillations, when using the LTS-D.

During our scenario the BMV group had ten seconds less time because there was no time needed for insertion of the LTS-D in this group. Those ten seconds were not the reason for the ability to give less than three shocks during the scenario in this group.

The ERC guidelines of 2005 recommends following alternatives for airway management [1]:

• laryngeal tube (LT).

• combitube (CT).

• laryngeal mask (LMA).

• LMA Fast Trach.

These are recommended as alternatives to endotracheal intubation, especially for those unfamiliar with endotracheal intubation and therefore for those who are not trained and experienced in this skill [1]. In most cardiac arrest situations using BMV is the first way to ventilate a patient because this equipment is immediately to hand. The ERC points out that BMV used during single rescuer resuscitation requires considerable skill. Therefore alternative airway devices seem to be necessary for airway management during cardiac arrest. This airway equipment should be immediately to hand.

The ERC defines ICU nurses as unfamiliar with endotracheal intubation (definition: personnel without extensive training and regular practice in endotracheal intubation [1]). Thus, during resuscitation they should refrain from attempting this measure, and restrict themselves to BMV as one method of airway management. The design of our study specifically took this into account. No participant was familiar in using supraglottic airway devices. Therefore the ICU nurses use BMV as the first method of airway management.

In our study BMV and LTS-D were the first methods of airway management. In this study on resuscitation manikins the LTS-D was superior to BMV when used by ICU nurses. The use of BMV does require a certain amount of experience, and in this context it is important to mention that only few study subjects had attended an ALS-course previously. This might explain why this group of qualified ICU nurses were so unsuccessful with BMV [9].

This leads to the conclusion that personnel unfamiliar with endotracheal intubation should receive better training the use of alternative devices. Concerning the ERC 2005 guidelines this fact should be integrated in the contents and structure of ALS courses [1].

Our findings that the LTS-D is easy to handle and seems to be a fast and reliable device for airway management are in accordance with previously published results on LT and LTS [2,10,11]. A 30 minute practice block on airway management using LTS-D enabled 96% of participants to place this airway device correctly on the first attempt. Participants consistently rated the LTS-D as an easy to handle tool, and they reported an improved sense of airway control. In this context the LTS-D may be a valuable first choice for airway management by personnel unfamiliar with BMV and endotracheal intubation. During a cardiac arrest situation there seems to be no contraindication for using the LTS-D. It is necessary to use the LTS-D according to published general instructions to avoid complications and to become familiar with this kind of airway device [12].

Since our data were obtained from simulated scenarios, drawing conclusions for actual resuscitation situations is difficult. Nevertheless, the LTS-D and its predecessor model have been used successfully in patients undergoing anaesthesia for surgery [11,13,14]. Comparing the other airway alternatives listed by the ERC, a survey of current publications shows the LT and the LT with suction option to be equally effective as other airway devices, like LMA, and Pro Seal LMA [11,13-17]. In routine use the LTS seems to be a better airway device than the combitube (CT) [11,17].

Studies investigating endpoints as well as survival with regard to the type of airway management employed are lacking.

A drawback of the LTS-D and other alternative airway devices is the fact that medication cannot be applied endobronchially. Since 2005, the ERC recommends intraosseous drug administration as most important alternative to the intravenous route in adults [1]. Thus, the lack of the endotracheal route using alternative airway devices is negligible.

Our participants were able to adhere to the ERC guidelines 2005 using the LTS-D. Because our data are derived from simulated scenarios, drawing conclusions for real resuscitation situations is difficult. Further studies in actual resuscitation situations to show a possible positive effect on patient's survival and neurological outcome are needed to confirm our preliminary simulator results.

One weakness of our study may be the fact that the LTS-D seems to be easier to insert in manikins, whereas BMV ventilation might be likely harder on Laerdal manikins than on real patients [18,19]. Therefore it is quite possible that the results bias in favour of the LTS-D and against BMV. This seems to be a phenomenon like in real patients, too. We mention that the results in a manikin study are not comparable with the use in real patients. Further studies in actual resuscitation situations are needed to confirm our preliminary simulation results.

Our data leads to the conclusion that ICU nurses unfamiliar with tracheal intubation account the use of an alternative type of airway device (e.g. LT). We advise that it is difficult to give clinical recommendations based on a manikin study.

## Conclusion

In this manikin study with single rescuer, we demonstrated that the LTS-D is an easy to handle alternative for airway management during resuscitation, especially for those unfamiliar with endotracheal intubation. Using the LTS-D in a manikin appears to be superior to BMV, as demonstrated in the reduction of NFT. In addition participants could only adhere to the time-frame of the ERC 2005 guidelines when using the LTS-D compared to BMV.

## Conflicts of interests

C Wiese and all co-authors declare: There are no conflicts of interest concerning any product or company which is mentioned in the study.

## Authors' contributions

CW: Design of study, Backgrounds and instruction of the course and scenario.

UB: Design of study, Statistical analysis.

AS: Statistical analysis, language correction.

TS: Instruction of the course and scenario, Result analysis.

AT: Instruction of the course and scenario and documentation of all results.

JB: Conceived of the study, and participated in its design and coordination.

BG: Study coordination and revise of the manuscript.

All authors read and approved the final manuscript.

## Pre-publication history

The pre-publication history for this paper can be accessed here:



## References

[B1] European Resuscitation Council (2005). European Resuscitation Council Guidelines for Resuscitation 2005. Resuscitation.

[B2] Agro F, Cataldo R, Alfano A, Galli B (1999). A new prototype for airway management in an emergency: the Laryngeal Tube. Resuscitation.

[B3] Asai T, Shingu K (2005). The laryngeal tube. Br J Anaesth.

[B4] Genzwuerker HV, Dhonau S, Ellinger K (2002). Use of the laryngeal tube for out-of-hospital resuscitation. Resuscitation.

[B5] Genzwuerker HV, Oberkinkhaus J, Finteis T, Kerger H, Gernotti C, Hinkelbein J (2005). Emergency airway management by first responders with the laryngeal tube – intuitive and repetive use in a manikin. Scand J Trauma Resusc Emerg Med.

[B6] Kette F, Reffo I, Giordani G, Buzzi F, Borean V, Cimarosti R, Codigla A, Hattinger C, Mongiat A, Tararan S (2005). The use laryngeal tube by nurses in out-of-hospital emergencies: Preliminary experience. Resuscitation.

[B7] Genzwuerker H, Finteis T, Hinkelbein J, Ellinger K (2003). First clinical experiences with the new LTS. A laryngeal tube with an oesophageal drain. Anaesthesist.

[B8] Salako SE (2006). The declaration of Helsinki 2000: ethical principles and the dignity of difference. Med Law.

[B9] Goedecke A, Keller C, Voelckel WG, Dünser M, Paal P, Torgersen C, Wenzel V (2006). Mask ventilation as an exit strategy of endotracheal intubation. Anaesthesist.

[B10] Genzwuerker HV, Hilker T, Hohner E, Kuhnert-Frey B (2000). The laryngeal tube: a new adjunct for airway management. Prehosp Emerg Care.

[B11] Bein B, Carstensen S, Gleim M, Claus L, Tonner PH, Steinfath M, Scholz J, Dörges V (2005). A comparison of the proseal laryngeal mask airway, the laryngeal tube S and the oesophageal-tracheal combitube during routine surgical procedures. Eur J Anaesthesiol.

[B12] Doerges V, Wenzel V, Schumann T, Neubert E, Ocker H, Gerlach K (2001). Intubating laryngeal mask airway, laryngeal tube, 1100 ml self-inflating bag – alternatives for basic life support?. Resuscitation.

[B13] Cook TM, Mc Cormick B, Asai T (2003). Randomized comparison of laryngeal tube with classic laryngeal mask airway for anaesthesia with controlled ventilation. Br J Anaesth.

[B14] Wrobel M, Grundmann U, Wilhelm W, Wagner S, Larsen R (2004). Laryngeal tube versus laryngeal mask airway in anaesthetised non-paralysed patients. A comparison of handling and postoperative morbidity. Anaesthesist.

[B15] Cook TM, Mc Kinstry C, Hardy R, Twigg S (2003). Randomized crossover comparison of the ProSeal laryngeal mask airway with the Laryngeal Tube during anaesthesia with controlled ventilation. Br J Anaesth.

[B16] Gaitini LA, Vaida SJ, Somri M, Yanovski B, Ben-David B, Hagberg CA (2004). A randomized controlled trial comparing the Pro Seal Laryngeal Mask Airway with the Laryngeal Tube Suction in mechanically ventilated patients. Anesthesiology.

[B17] Bein B, Scholz J (2005). Supraglottic airway devices. Best Prac Res Clin Anaesthesiol.

[B18] Cook TM, Bayley G, Jordan G, Silsby J (2007). A comparison of four different advanced airway mannequins for training DAS guidelines. Anaesthesia.

[B19] Jackson KM, Cook TM (2007). A comparison of four different advanced airway mannequins for training SAD insertion. Anaesthesia.

